# Diesel Particulate Matter Induces Receptor for Advanced Glycation End-Products (RAGE) Expression in Pulmonary Epithelial Cells, and RAGE Signaling Influences NF-κB–Mediated Inflammation

**DOI:** 10.1289/ehp.1002520

**Published:** 2010-11-18

**Authors:** Paul R. Reynolds, Karisa M. Wasley, Camille H. Allison

**Affiliations:** Department of Physiology and Developmental Biology, Brigham Young University, Provo, Utah, USA

**Keywords:** diesel, inflammation, lung, NF-κB, RAGE

## Abstract

**Background:**

Receptors for advanced glycation end-products (RAGE) are cell-surface receptors expressed by alveolar type I (ATI) epithelial cells and are implicated in mechanisms of alveolar development and sustained pulmonary inflammation.

**Objectives:**

In the present study, we tested the hypothesis that diesel particulate matter (DPM) up-regulates RAGE in rat ATI-like R3/1 cells and human primary small airway epithelial cells (SAECs), leading to an inflammatory response.

**Methods and Results:**

Using real-time reverse transcriptase polymerase chain reaction and immunoblotting, we found that *RAGE* mRNA and protein are up-regulated in cells exposed to DPM for 2 hr. Use of a luciferase reporter containing nuclear factor-κB (NF-κB) response elements revealed decreased NF-κB activation in cells transfected with small interfering RNA (siRNA) for RAGE (siRAGE) before DPM exposure compared with cells transfected with scrambled control siRNA (siControl). In addition, immunostaining revealed diminished nuclear translocation of NF-κB in DPM-exposed cells transfected with siRAGE compared with cells transfected with siControl before DPM stimulation. Enzyme-linked immunosorbent assay demonstrated that in R3/1 cells DPM induced secretion of monocyte chemoattractant protein-1 (MCP-1) and interleukin-8 (IL-8), two cytokines induced by NF-κB and associated with leukocyte chemotaxis during an inflammatory response. Incorporating siRAGE was sufficient to significantly decrease DPM-induced MCP-1 and IL-8 secretion compared with cells transfected with siControl.

**Conclusions:**

These data offer novel insights into potential mechanisms whereby RAGE influences pulmonary inflammation exacerbated by DPM exposure. Further research may demonstrate that molecules involved in RAGE signaling are potential targets in lessening the degree of particulate matter-induced exacerbations of inflammatory lung disease.

Diesel particulate matter (DPM) comprises a collection of minute substances generated by vehicular traffic that contribute substantially to particulate matter (PM) air pollution characteristic of urban areas. The diameter of DPM is directly related to its biological properties, and PM, including DPM, with aerodynamic diameters ≤ 10 and 2.5 μm are classified as PM_10_ and PM_2.5_, respectively (Churg and Brauer 1997). Although PM_10_ can penetrate deep into the respiratory tree, PM_2.5_ can easily reach alveolar parenchymal cells and can be internalized by alveolar epithelium and macrophages (Churg and Brauer 1997; Kim et al. 1994). Inhalation of DPM has been associated with a host of cardiovascular and respiratory diseases, such as asthma, chronic obstructive pulmonary disease (COPD), and pulmonary fibrosis, which all contribute to significant morbidity and mortality (Bayram et al. 2006; Dockery et al. 1993; Hales et al. 2000; Harre et al. 1997). Of note, epidemiological studies have revealed remarkable associations between PM content in ambient air and increased rates of respiratory disease in susceptible populations (Dockery et al. 1993; Pope et al. 1995). Despite this current knowledge, it remains unclear which components in PM air pollution are responsible for adverse respiratory effects and which mechanisms are directly involved (Dreher 2000).

Receptors for advanced glycation end-products (RAGE) are members of an immunoglobin superfamily of cell-surface proteins expressed by many cell types, including smooth muscle cells, fibroblasts, macrophages and monocytes, and epithelial cells (Demling et al. 2006; Thornally 1998). RAGE expression is most abundant in well-differentiated alveolar type I (ATI) cells in the lung (Schmidt et al. 2001). Identification in alveolar epithelial cells has led to the implication of RAGE in important developmental processes such as morphological differentiation and increased adherence that characterize the transitioning of cuboidal surfactant-secreting ATII cells to squamous ATI cells (Buckley and Ehrhardt 2010). RAGE was first described as a transmembrane protein that acts as a progression factor in cellular responses induced by advanced glycation end-products (AGEs) that accumulate in hyperglycemia and oxidant stress (Schmidt et al. 1992). AGEs are stable chemical entities generated when simple sugars form amide linkages with amines on proteins, with further oxidant-induced molecular rearrangement via Maillard chemistry. The result is a group of chemical structures that can bind and activate RAGE (Schmidt et al. 1992). Other studies have identified endogenous ligands such as cytokine-like mediators of the S100/calgranulin family of calcium-binding proteins, amyloid β-peptide, and HMGB-1 (high mobility group box 1, or amphoterin). These ligands orchestrate changes in gene expression via a host of activated signal transduction pathways (Hofmann et al. 1999; Taguchi et al. 2000; Yan et al. 1996). Research to date culminates in the characterization of RAGE as a pattern recognition receptor capable of recognizing and binding a collection of molecules with variable yet related geometry.

RAGE expression increases as its ligand availability elevates (Schmidt et al. 2001), and RAGE-ligand interaction leads to pathological processes, including those associated with diabetic complications, neurodegenerative disorders, atherosclerosis, and inflammation (Hofmann et al. 1999; Taguchi et al. 2000). Despite known instances where RAGE is up-regulated in disease, the full extent of RAGE expression and the molecular mechanisms that regulate its expression and subsequent downstream effects have not been adequately evaluated. Understanding the potential role of RAGE in the context of PM exposure could provide insights into the mechanisms of PM-induced pulmonary inflammation and provide opportunities for the reduction of PM-induced exacerbations common to chronic lung disease (Dockery et al. 1993; Ling and van Eden 2009; Schwartz 1995).

In the present study, we tested the hypothesis that pulmonary epithelial cells induce RAGE after exposure to DPM, a specific constituent of air pollution PM. We also tested the hypothesis that RAGE is directly involved in generating a proinflammatory state in epithelial cells after DPM exposure. Using rat R3/1 cells, an immortalized ATI cell line, and human primary distal airspace epithelium, we demonstrated that RAGE is up-regulated after exposure to DPM and that nuclear factor-κB (NF-κB)–mediated cytokine secretion occurs via RAGE signaling. Collectively, these data offer novel insights into potential mechanisms whereby RAGE influences inflammation in pulmonary epithelial cells after exposure to DPM.

## Materials and Methods

### Cell culture and DPM

R3/1 cells kindly provided by M. Kasper (Dresden University, Dresden, Germany) are derived from rat and are characteristic of ATI cells (Koslowski et al. 2004). R3/1 cells were maintained in Dulbecco’s modified Eagle’s medium (DMEM) supplemented with 10% fetal calf serum (FCS), l-glutamine, and penicillin-streptomycin (Sigma, St. Louis, MO). Small airway epithelial cells (SAECs; Lonza Inc., Walkersville, MD) are human primary pulmonary epithelial cells isolated from distal airspaces; they were maintained in saline-adenine-glucose-mannitol medium supplemented according to the supplier’s instructions. Cells were grown to 80–90% confluence and exposed to media supplemented with DPM or media alone for 2 hr. At the termination of the experiment, cells were treated in one of the following ways: immediately fixed for immunocytochemistry, lysed before RNA isolation for assessment by real-time reverse transcriptase polymerase chain reaction (RT-PCR), or lysed and subjected to immunoblot analysis.

DPM used in these experiments is cataloged at the National Institute of Standards and Technology (NIST) as Standard Reference Material (SRM) 2975; DPM used to prepare SRM 2975 (M.E. Wright, Donaldson Company, Inc., Minneapolis, MN) was collected from a filtering system designed specifically for diesel-powered forklifts (Wright et al. 1991). DPM was homogenized and extracted for preparation of SRM 2975 and SRM 1975 (National Institute of Standards and Technology 2000). DPM-supplemented cell culture medium was prepared by adding appropriate weights of DPM to phosphate-buffered saline (PBS). The resulting suspension was vigorously vortexed just before being added to freshly prepared DMEM plus 10% FCS media. Preliminary studies included DPM-supplemented media at concentrations that ranged from 1 to 50 μg/mL. The experiments described in the present investigation all involved exposure to 3 μg/mL DPM for 2 hr to determine DPM effects in ambient settings.

### RNA isolation and assessment by real-time RT-PCR

Total RNA was isolated from R3/1 cells using the Absolutely RNA RT-PCR Miniprep Kit (Stratagene, La Jolla, CA). After total RNA was spectrophotometrically quantified, reverse transcription and PCR amplification using a One-step Brilliant SYBR Green quantitative RT-PCR master mix kit (Stratagene) were performed in a single reaction following the manufacturer’s instructions. cDNA conversion, amplification, and data analysis were performed using a Mx3000P real-time PCR system computerized cycler (Stratagene). The following primers available through Primer Bank (ID no. 6671525a3) were synthesized and high-performance liquid chromatography purified by Invitrogen Life Technologies (Carlsbad, CA): *RAGE* (forward, ACT ACC GAG TCC GAG TCT ACC; reverse, GTA GCT TCC CTC AGA CAC ACA) and glyceraldehyde 3-phosphate dehydrogenase gene (*GAPDH*; forward, TAT GTC GTG GAG TCT ACT GGT; reverse, GAG TTG TCA TAT TTC TCG TGG). Primers were used at a concentration of 75 nM each in 25-μL reactions. Cycle parameters included 40 min at 55°C for reverse transcription and then 10 min at 95°C and 40 cycles of 30 sec at 95°C, 1 min at 58°C, and 30 sec at 72°C. Control samples lacking template or RT were included to identify primer-dimer products and to exclude possible contaminants. Data are presented as percent change normalized to *GAPDH*.

### Protein isolation and immunoblot analysis

Total protein was isolated from R3/1 cells using RIPA buffer and associated protease inhibitors (Santa Cruz Biotechnology, Santa Cruz, CA). Protein concentrations were determined by bicinchoninic acid assay to ensure equal loading for assessment by sodium dodecyl sulfate–polyacrylamide gel electrophoresis (SDS-PAGE). Briefly, 20 μg total protein was separated by SDS-PAGE, transferred to a nitrocellulose membrane, and blocked with 5% nonfat milk. The membrane was incubated with a primary RAGE polyclonal antibody (R&D Systems, Minneapolis, MN) diluted at 1:1,000 at 4°C overnight. Exposure to HRP-conjugated secondary antibodies (Thermo Fisher Scientific, Rockford, IL) was followed by development with electrochemiluminescence (Amersham Biosciences, Buckinghamshire, UK). Immunoblotting images presented here are representative of three experiments, and densitometric evaluation of the bands was conducted using UN-SCAN-IT gel densitizing software (Silk Scientific, Orem, UT). Density data were standardized to small interfering control RNA (siControl)–transfected cells and set to 1.

### Immunohistochemistry

Cells in select experiments were fixed by incubation in 4% paraformaldehyde for 20 min and then washed in three changes of PBS before assessment of active NF-κB by immunocytochemistry. Staining was conducted as generally outlined (Reynolds et al. 2008) by using a primary antibody against active NF-κB (NF-κB p65 monoclonal antibody; Cell Signaling, Beverly, MA) at a concentration of 1:50 and a donkey anti-rabbit secondary antibody (Santa Cruz). Control cultures were incubated in blocking serum alone. Individuals blinded to the type of small interfering RNA (siRNA) transfected and the presence or absence of DPM performed three counts of 100 cells in each experiment. Cells with > 50% immunoreactivity in the cytoplasm or nucleus were considered cytoplasmic or nuclear positive for NF-κB, respectively. Cells with an equal cytosolic/nuclear distribution of NF-κB were not considered. Total cells from the three counts were averaged, and standard deviations were determined.

### siRNA and vector transfection and reporter gene assays

As outlined in select experiments, R3/1 or SAECs were transfected with rat or human siRNA for *RAGE* (siRAGE) or a scrambled control siRNA sequence (siControl) generated by Santa Cruz 24 hr before DPM exposure. siRNA transfections were performed using the recommended transfection reagent mix (Santa Cruz). Functional assays of reporter gene constructs were performed by transient transfection of R3/1 cells grown to 40–50% confluence. Cells were transfected with 500 ng pRSV-βgal to determine transfection efficiency and 100 ng pNF-κB–Luc vector (Stratagene) or pcDNA control vector to bring total DNA concentration to 600 ng. Cells were allowed to grow 24 hr before exposure to DPM or fresh medium replacement. After 2 hr of DPM exposure, cells were washed and lysed, and cleared supernatant was used for both β-gal and luciferase assays. Reporter assays were normalized for transfection efficiency based on β-gal assays performed as previously described (Reynolds et al. 2004). Luciferase activity was determined in 10 μL extract at room temperature with 100 μL luciferase reagent (Promega Corporation, Madison, WI) for 10 sec after a 2-sec delay in a Monoight 3010 luminometer (BD Biosciences, Franklin Lakes, NJ).

### Measurement of cytokine levels

Enzyme-linked immunosorbent assay (ELISA) was used to assess the concentration of cytokines secreted by R3/1 cells. Briefly, media were removed before cell lysis, and equal volumes of cell culture media were assessed in each experimental group for concentrations of monocyte chemoattractant protein-1 (MCP-1; 100 μL/sample) and interleukin-8 (IL-8; 50 μL/sample) in triplicate using a rat MCP-1 ELISA kit (Ray Biotech, Norcross, GA) or a Quantikine Rat CXCL1/CINC-1 ELISA kit (R&D Systems) as directed by the manufacturer.

### Statistical analysis

Values are expressed as mean ± SD obtained from at least three separate experiments in each group. Data were assessed by one- or two-way analysis of variance (ANOVA). When ANOVA indicated significant differences, the Student *t*-test was used with Bonferroni correction for multiple comparisons. Results presented are representative, and those with *p*-values < 0.05 were considered significant. We used S-Plus 8 (Tibco Software, Palo Alto, CA) to analyze the data.

## Results

### DPM induces RAGE mRNA and protein in R3/1 cells and SAECs

**Figure 1:**
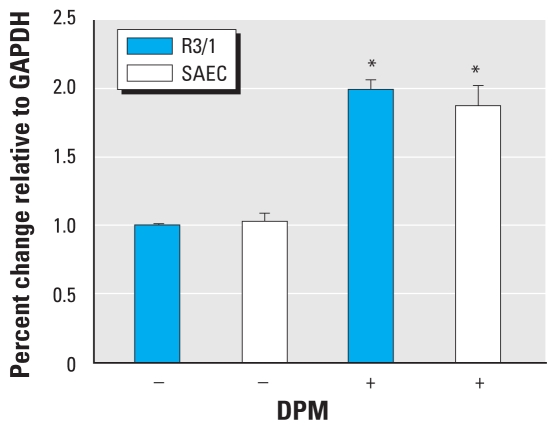
*RAGE* mRNA was induced by DPM in R3/1 cells and SAECs. Quantitative real-time RT-PCR revealed a significant increase in *RAGE* mRNA expression in R3/1 cells and SAECs exposed to 3 μg/mL DPM for 2 hr compared with unstimulated control cells. Experiments were performed in triplicate. Abbreviations: +, with; −, without. *p ≤ 0.05.

**Figure 2:**
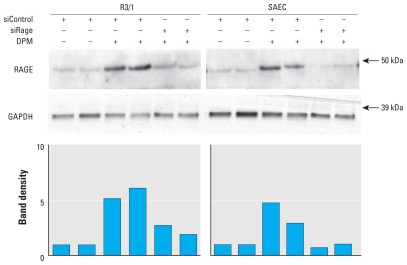
RAGE protein was induced by DPM in R3/1 cells and SAECs. Immunoblotting for RAGE revealed a marked increase in RAGE protein synthesis in cells transfected with siControl 24 hr before a 2 hr exposure to 3 μg/mL DPM. Densitometry of bands revealed clear reductions in DPM-induced RAGE expression when siRAGE was transfected 24 hr before DPM exposure. Abbreviations: +, with; −, without; kDa, kilodaltons.

To determine whether RAGE is up-regulated by exposure to DPM, we assessed *RAGE* mRNA levels in R3/1 cells and SAECs by quantitative real-time RT-PCR and compared cells exposed to DPM or fresh media. Compared with cells grown in culture media alone, exposure to DPM for 2 hr induced a significant 100% increase in *RAGE* mRNA expression (Figure 1). To determine possible correlation between *RAGE* mRNA and protein expression, we performed immunoblotting to evaluate relative concentrations of RAGE protein. Immunoblot analysis revealed an anticipated augmentation in RAGE protein expression in freshly lysed cells exposed to DPM for 2 hr compared with cells grown in the absence of DPM (Figure 2). Furthermore, RAGE was diminished in cells that were transfected with siRAGE before DPM exposure (Figure 2).

### RAGE contributes to DPM-induced NF-κB activity

**Figure 3:**
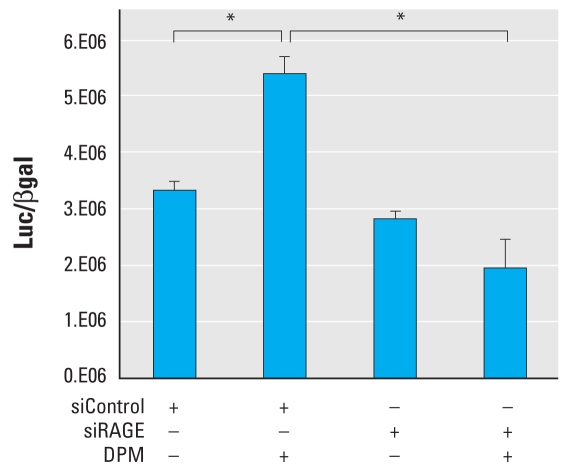
RAGE inhibition by siRNA blocked activation of NF-κB by DPM in R3/1 cells. R3/1 cells were transfected with an NF-κB–Luc vector and siRAGE or siControl 24 hr before DPM exposure or fresh medium replacement. Exposure to DPM for 2 hr significantly induced nuclear NF-κB activity in siControl-transfected R3/1 cells. When siRAGE was incorporated, DPM-induced NF-κB activity was markedly decreased to below unstimulated basal levels. Abbreviations: +, with; −, without. *p ≤ 0.05.

Because respiratory epithelial cells exposed to DPM resulted in significant RAGE up-regulation, we sought to uncover possible downstream signaling events in cells with increased RAGE. Accordingly, we performed gene reporter experiments to assess NF-κB activity in R3/1 cells exposed to DPM. Exposure to DPM 24 hr after transfection of NF-κB–luciferase reporters revealed that R3/1 cells initiate significant nuclear translocation of NF-κB compared with cells without DPM exposure (Figure 3). Additional experiments revealed that cells transfected with siRAGE before DPM stimulation experienced complete inhibition of NF-κB activity attributed to DPM exposure. Specifically, nuclear translocation and activation of NF-κB in DPM-exposed siRAGE transfected cells were not significantly different from siControl-transfected cells maintained without DPM exposure (Figure 3).

To further investigate the significance of RAGE abrogation by siRNA and resulting activation and nuclear translocation of NF-κB after DPM exposure, we performed immunostaining for active NF-κB (data not shown). In cells transfected with siControl, immunostaining revealed prominent cytosolic localization of NF-κB; however, stimulation of siControl-transfected R3/1 cells with DPM resulted in marked nuclear localization of NF-κB. When we transfected cells with siRAGE before DPM exposure, we detected sequestration of NF-κB in the cytoplasm compared with siControl transfected cells incubated with DPM. A no primary staining control (staining without NF-κB antibody) demonstrated antibody immunospecificity. Assessment of prominent cytoplasmic versus nuclear NF-κB immunostaining via randomized cell counts revealed that siRAGE prevented significant DPM-induced NF-κB translocation to the nucleus (Table 1).

### DPM-induced proinflammatory cytokine secretion is mediated by RAGE

**Figure 4:**
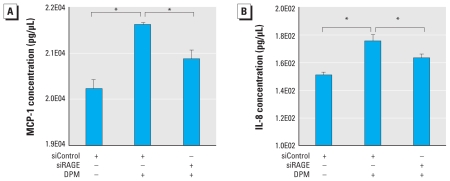
DPM-induced secretion of MCP-1 and IL-8 was mediated by RAGE. (*A*) ELISAs demonstrate that R3/1 cells exposed to DPM for 2 hr significantly increased MCP-1 secretion. Compared with control cells exposed to DPM, siRAGE incorporation significantly decreased DPM-induced MCP-1 secretion. (*B*) IL-8 was significantly up-regulated by R3/1 cells after DPM exposure, and secretion of IL-8 was significantly diminished in cells previously transfected with siRAGE. Abbreviations: +, with; −, without. *p ≤ 0.05.

After the discovery that nuclear translocation of active NF-κB in R3/1 cells exposed to DPM is mediated at least in part by RAGE, we designed additional experiments to evaluate cytokine secretion. The addition of DPM to R3/1 cells for 2 hr resulted in increased secretion of MCP-1 and IL-8, two proinflammatory cytokines secreted by epithelial cells and known transcriptional targets of NF-κB (Figure 4). Notably, cells transfected with siRAGE before DPM exposure resulted in significantly diminished MCP-1 and IL-8 elaboration compared with siControl-transfected cells exposed to DPM.

## Discussion

### DPM up-regulates RAGE mRNA and protein

Research performed previously in this laboratory revealed that RAGE functions during PM exposure. Specifically, data demonstrated that cigarette smoke PM causes significant induction of both RAGE and Egr-1 (early growth response gene-1), a zinc-finger–containing transcription factor that influences cell responses to stimuli including cytokines, apoptosis-promoting factors, and injury (Beckmann and Wilce 1997; O’Donovan et al. 1999). Additional research clarified transcriptional control of *RAGE* by Egr-1 and the likelihood of a profound positive feedback loop wherein smoke-induced *RAGE* expression is further augmented by transcriptional up-regulation of the receptor by Egr-1 (Reynolds et al. 2006). Because there is a clear function for RAGE in cigarette-smoke–related PM exposure, we hypothesized that the role of RAGE as a pattern recognition receptor also includes the orchestration of inflammation resulting from other forms of fine PM, such as DPM.

The discovery that DPM induces *RAGE* mRNA and protein in immortalized alveolar epithelium is, by nature, associated with a restriction in extrapolation to *in vivo* human settings. However, similar observations in human SAECs suggest that RAGE up-regulation is likely associated with cellular responses to DPM exposure. Although conventional thought stipulates that fresh DPM must contain ultrafine organic PM, including polycyclic aromatic hydrocarbons and quinones, to be biologically active (Wichmann 2007), our data suggest that aged DPM is not inert but is capable of biological activity. Because aged DPM is abundantly suspended in the atmosphere, these studies provide important observations from the standpoint of public health. Evidence further suggests that organic compounds associated with DPM are capable of adverse effects via redox reactions, culminating in the generation of intracellular reactive oxygen species (Li et al. 2003; Shinyashiki et al. 2009). Furthermore, because ligand prevalence induces RAGE expression (Schmidt et al. 2001), RAGE activation by DPM may also occur because of the presence of AGE moieties on combusted DPM. The chemistry associated with the burning of cigarettes leads to the formation of AGE products detected in cigarette smoke (Cerami et al. 1997; Dickerson and Janda 2002; Nicholl and Bacala 1998). Although it has not been fully explored, it is likely that diesel combustion, like the burning of tobacco, will also produce oxidation products and stable AGEs because of the facilitation of AGE chemistry by lower temperatures attributed to diesel engines.

Although the correlation between DPM inhalation and pulmonary inflammation is well established (Salvi et al. 2000), understanding the intricate mechanisms involved in perpetuating DPM-mediated inflammatory responses has been enigmatic. Because inhalation of DPM directly exposes airway and alveolar epithelium to harmful constituents, understanding the biology of stimulated epithelial cells, a significant source of proinflammatory mediators, is essential. RAGE up-regulation by DPM may provide additional receptors necessary in first-response mechanisms associated with epithelial cells positioned at the interface between inhaled air and lung parenchyma. Recent research supporting the concept of increased receptor availability considers the role of RAGE and its ligands in the effects of exposure to PM derived by fossil fuel combustion and the possible exacerbation of debilitating pulmonary disorders such as asthma and COPD (Campo et al. 2008; Ferhani et al. 2010; Halayko and Ghavami 2009).

### DPM induces RAGE-mediated activation of NF-κB and cytokine secretion

In cells with differentially regulated RAGE expression, we assessed NF-κB after DPM exposure to evaluate a proposed RAGE-mediated proinflammatory pathway. NF-κB was initially identified as a transcription factor in B cells and has since been ubiquitously detected in the cytoplasm of all cell types (Aggarwal 2004). When activated by one of a host of different stimuli, NF-κB translocates to the nucleus, where it regulates the expression of more than 200 genes that influence cell growth, survival, and inflammation (Aggarwal 2004). Although an association between DPM exposure and the activation of factors such as NF-κB and activator-protein-1 (a transcription factor) has been established (Takizawa et al. 1999), the present investigation supports the notion that increased availability of RAGE is important in DPM-mediated NF-κB activation during an inflammatory response. Despite this novel discovery, additional research that evaluates acute pathways of DPM-mediated pulmonary inflammation initiated by RAGE ligation is necessary. Such a comprehensive study requires assessment of RAGE and subsequent intracellular signal transduction involving NF-κB and its upstream stress-related kinases, including mitogen-activated protein kinases, p38, and c-jun N-terminal kinases (Dourazar et al. 2005).

Our studies revealed increases in the synthesis and secretion of IL-8 and MCP-1 by alveolar epithelium after exposure to DPM. IL-8 is a CXC chemokine that predominantly influences neutrophil chemotaxis. Because of its remarkable stability, IL-8 can maintain prolonged biological activity despite changes in physiological homeostasis (Baggiolini and Clark-Lewis 1992). MCP-1 is a small cytokine belonging to the CC family of chemokines that activates monocytes, lymphocytes, mast cells, eosinophils, and basophils (Oppenheim et al. 1991). The present data reveal that, compared with nonexposed controls, RAGE targeting by siRAGE cannot alone completely inhibit DPM-induced IL-8 and MCP-1 secretion. However, a significant reduction in DPM-induced cytokine elaboration by cells with diminished RAGE compared with control cells exposed to DPM remains noteworthy. These findings strongly suggest that RAGE signaling is important in the release of inflammatory mediators but that other factors and pathways are also involved. Despite significant RAGE-mediated reductions in the inflammatory status of epithelial cells, the complete inflammatory profile of cells exposed to DPM is likely maintained by a complex array of molecules functioning in different yet parallel pathways.

Although the amplitude of IL-8 and MCP-1 induction by DPM and the degree of prevented cytokine secretion by siRAGE transfected cells were not extremely large, individual replicates were tightly grouped and reproducible. It remains plausible that secretion of IL-8 and MCP-1 after only 2 hr of DPM exposure serves as an initial response necessary in the activation of subsequent intermediates in inflammatory cascades. Continuous release of IL-8 and MCP-1, even at very low levels, could also assist in the recruitment of additional immune-competent cells such as lymphocytes and macrophages. Additional inflammatory mediators may then lead to the amplification of the initial trigger commenced by IL-8, MCP-1, and other early modulators. An example of this potential paradigm is apparent in studies that analyze bronchoalveolar lavage (BAL) fluid in patients with chronic bronchitis. BAL fluid obtained from affected individuals revealed that MCP-1 levels are significantly elevated compared with healthy controls despite the observation that initial MCP-1 induction is modest (Capelli et al. 1999). It is likely that high concentrations of MCP-1 and other early cytokines are not necessary when recruiting competent immune cells capable of profound proinflammatory effects.

## Conclusions

In this study we report additional biological information regarding RAGE-mediated signaling pathways activated by DPM, a near ubiquitous, pervasive environmental contaminant. Here we provide evidence that alveolar epithelial cells exposed to DPM significantly increased *RAGE* mRNA and protein synthesis. Furthermore, DPM-induced activation of NF-κB and the secretion of two NF-κB targets, IL-8 and MCP-1, were significantly influenced by RAGE signaling. Together, these data reveal for the first time that RAGE signaling may be involved in inflammatory responses triggered by DPM exposure. Although we demonstrate that RAGE targeting blocks DPM-induced NF-κB activity, understanding the role and efficacy of RAGE and other parallel pathways in attenuating inflammatory responses elicited by DPM is critically necessary. Currently, important extensions of this research seek to evaluate the role of RAGE in other pulmonary cell types and mouse models exposed to DPM. Further studies may demonstrate that RAGE is a possible target in the successful pharmacological treatment of DPM-exacerbated chronic lung diseases such as COPD and asthma.

## Figures and Tables

**Table 1:** Percentage of cells with compartmentalized NF-κB localization (mean ± SD).

Compartment	siControl	siControl + DPM	siRAGE	siRAGE + DPM
Percent cytoplasmic	89.3 ± 8.9	43.8 ± 8.5	83.6 ± 6.3	62.6 ± 5.7*
Percent nuclear	12.6 ± 5.2	58.3 ± 4.8	19.7 ± 7.3	41.5 ± 6.2*

* *p* < 0.05 compared with siControl + DPM.
